# Tenascin-C in Heart Diseases—The Role of Inflammation

**DOI:** 10.3390/ijms22115828

**Published:** 2021-05-29

**Authors:** Kyoko Imanaka-Yoshida

**Affiliations:** 1 Department of Pathology and Matrix Biology, Mie University Graduate School of Medicine, Tsu, Mie 514-8507, Japan; imanaka@doc.medic.mie-u.ac.jp; 2 Mie University Research Center for Matrix Biology, Mie University Graduate School of Medicine, Tsu, Mie 514-8507, Japan

**Keywords:** inflammation, myocardial infarction, hypertensive fibrosis, myocarditis, dilated cardiomyopathy, remodeling, autoimmunity, viral infection

## Abstract

Tenascin-C (TNC) is a large extracellular matrix (ECM) glycoprotein and an original member of the matricellular protein family. TNC is transiently expressed in the heart during embryonic development, but is rarely detected in normal adults; however, its expression is strongly up-regulated with inflammation. Although neither TNC-knockout nor -overexpressing mice show a distinct phenotype, disease models using genetically engineered mice combined with in vitro experiments have revealed multiple significant roles for TNC in responses to injury and myocardial repair, particularly in the regulation of inflammation. In most cases, TNC appears to deteriorate adverse ventricular remodeling by aggravating inflammation/fibrosis. Furthermore, accumulating clinical evidence has shown that high TNC levels predict adverse ventricular remodeling and a poor prognosis in patients with various heart diseases. Since the importance of inflammation has attracted attention in the pathophysiology of heart diseases, this review will focus on the roles of TNC in various types of inflammatory reactions, such as myocardial infarction, hypertensive fibrosis, myocarditis caused by viral infection or autoimmunity, and dilated cardiomyopathy. The utility of TNC as a biomarker for the stratification of myocardial disease conditions and the selection of appropriate therapies will also be discussed from a clinical viewpoint.

## 1. Introduction

Tenascins are a family of large extracellular matrix (ECM) glycoproteins comprising 4 members: tenascin-C, R, X, and W [1,2,3,4,5]. Tenascin-C (TNC) was the first tenascin to be identified [6] and is the most characterized member of this family (reviewed in [1,7,8,9]). It is also an original member of the ‘matricellular protein’ family together with thrombosondin-1 and SPARC (secreted protein acidic and rich in cysteine; osteonectin) [10,11]. Matricellular proteins are a growing group of non-structural ECM proteins with the following common unique properties: (1) they are strongly up-regulated during embryonic development and tissue remodeling after injury; (2) they serve as biological mediators of cell function by binding to various cell surface receptors, other ECM molecules, or growth factors; and (3) they often induce de-adhesion or counter-adhesion.

One of the characteristics of TNC as a typical matricellular protein is that it is transiently expressed at specific sites during embryogenesis, weakly expressed in normal adults, and up-regulated under pathological conditions in various tissues. This spatiotemporally limited expression pattern is particularly prominent in the heart (reviewed in [12,13]). Accumulating evidence has shown diverse and important roles for TNC in responses to injury as well as cancer stroma [14,15], particularly in the regulation of inflammation [16,17]; however, neither the knockout nor overexpression of TNC induces a distinct phenotype during heart development [18,19].

The significant role of inflammation in the pathophysiological processes of various heart diseases has been attracting attention [20,21] since elevated levels of tumor necrosis factor (TNF) were reported in patients with heart failure for the first time in 1990 [22]. Various infectious pathogens or environmental injuries, such as ischemia and hemodynamic overloading, induce different types of inflammatory/immune responses. Inflammation is a response that facilitates adaptation to abnormal conditions and restores homeostasis and cardiovascular function. However, dysregulated inflammation leads to collateral myocardial damage that ultimately results in progressive ventricular dysfunction and adverse ventricular remodeling.

This review will primarily focus on the roles of TNC in various inflammatory reactions in heart diseases, including myocardial infarction (MI), pressure overload fibrotic hearts, and myocarditis/dilated cardiomyopathy (DCM) from a clinical viewpoint.

## 2. General Features of TNC in the Heart

TNC is transiently expressed during embryonic heart development and is rarely detected in normal adults, but is re-expressed in diseased hearts (reviewed in [12,13]).

Precardiac mesodermal cells, a special population of cardiomyocytes in embryonic hearts [18], and several cell lines of cardiomyocytes [23,24] have the potential to produce TNC. However, a major source of TNC in the adult pathological heart is residential interstitial fibroblasts [25,26,27]. A number of cardiac remodeling factors, including proinflammatory cytokines/chemokines, growth factors, angiotensin II [28], and endothelin I [29], up-regulate the expression of TNC in cardiac fibroblasts [12,30]. Multiple signaling cascades, including TGF-β/Smad 3/4, Toll-like receptor 4 (TLR4)/NFkB, PDGF/phosphoinositide 3-kinase/Akt, and PDGF/MAPK, may modulate the expression of TNC [2,31,32,33].

## 3. Embryonic Heart Development and TNC

The transient expression of TNC is often associated with cell migration and epithelial–mesenchymal/mesenchymal–epithelial transition (EMT/MET) in the following important steps of cardiac morphogenesis: (1) the differentiation of cardiomyocytes from the mesoderm, (2) cushion tissue and valve formation, and (3) coronary vascular development.

Precardiac mesodermal cells in the first heart field (FHH) express TNC in association with MET; however, its expression is quickly down-regulated when cells differentiate to express sarcomeric proteins. Cardiomyocytes originating from the secondary heart field in the outflow tract maintain the expression of TNC during the looping and shortening of the primitive heart tube [18].

During the complex morphogenesis of the four-chambered heart, endocardial cells, which form cushion tissue to develop the cardiac septa and valves, express TNC in association with EMT [18]. TNC is expressed at important steps during coronary vasculogenesis. Progenitor cells of the coronary vessels originate from the proepicardial organ (PEO) (reviewed in [34,35]) and migrate along the surface of the primitive heart tube to form the epicardium, which undergoes EMT to give rise to the coronary vasculature and interstitial fibroblasts. TNC is expressed in cells in PEO and its expression is associated with epicardial EMT [18].

A primitive vascular network forms to cover the surface of the ventricle, which is then remodeled and connected to the aorta to form the proximal region of coronary arteries [36,37,38]. During this process, TNC appears to guide developing vascular channels due to its spatial relationship: the vascular network is not formed in the TNC-positive outflow tract and eventually reaches the base of the aorta with the shortening of the TNC-positive outflow tract. TNC is also up-regulated with the maturation of coronary arteries and promotes the recruitment of vascular mural cells through PDGF-B-B/PDGF receptor β [39,40,41]. Although this strictly limited spatio-temporal expression suggests essential roles for TNC during embryonic heart development, its deletion or overexpression does not induce distinct changes in cardiogenesis [18,19].

Two different lines of TNC-deficient mice have been generated [42,43] and neither show a distinct morphological phenotype. However, careful analyses demonstrated that a deficiency of TNC may induce minor differences in the organogenesis of the prostate gland [44] and lungs [45,46], behavioral abnormalities [47,48,49], and pulmonary functional dysfunction in adulthood [50]. In comparisons with wild-type mice, no prominent differences were detected in cardiac morphology, histology, and function or in blood pressure in adult TNC-null mice [23,51,52,53,54,55,56,57] or in TNC-overexpressing mice [19].

## 4. Myocardial Infarction

### 4.1. Acute Myocardial Infarction and TNC

MI is the death of a part of the myocardium due to prolonged severe ischemia caused by coronary arterial occlusion, which induces typical acute sterile inflammation and subsequent myocardial repair.

In the early stage after infarction, at approximately 1–3 days, matrix metalloproteinases (MMPs) are activated and generate the fragmentation of ECM proteins to form a provisional matrix, which serves as a scaffold for cell adhesion. At approximately 3–7 days, inflammatory cells, primarily macrophages, actively remove necrotic myocytes (the inflammatory phase). Between 1–2 weeks, the damaged area is progressively replaced by the ingrowth of highly vascularized granulation tissue. Inflammatory cells are cleared, followed by the migration and proliferation of fibroblasts/myofibroblasts and vascular cells (the proliferative phase). The density of myofibroblasts is reduced and various matrix molecules in the infarct area interact and form mature collagen fibers, leading to scar tissue (the maturation phase) [13].

The expression of TNC becomes detectable within 24 h of the onset of infarction in experimental animal models, peaks on days 3–5, and disappears by day 28 [25,54,58]. Serum levels of TNC in human patients reflect this time course of expression [59]. TNC is exclusively expressed at the border zone between an infarct lesion and the intact myocardium [25,54,57,60,61].

The timing and location of the expression of TNC suggest that it plays special roles in the inflammatory phase to the early proliferative phase after MI. Several roles have been described for TNC during tissue repair after MI.

As a typical “de-adhesion” molecule (reviewed in [7,62]), TNC may loosen the adhesion of surviving cardiomyocytes from the surrounding connective tissue. In the normal heart, cardiomyocytes firmly adhere to the surrounding connective tissue via a costamere adhesion complex [63,64,65]. Although TNC does not break this strong adhesion by itself [25], it may free cardiomyocytes by promoting the expression of MMP-2 and MMP-9 [23], thereby facilitating the movement of cells for rearrangement. TNC simultaneously adheres weakly to cardiomyocytes, similar to a ‘post it’ [25,30], and may protect against anoikis.

TNC may also protect cardiomyocytes as a shock absorber. The border zone may be exposed to pathological mechanical stress due to a difference in the physical properties of the intact myocardium and necrotic tissue [66]. An adaptive and protective role for TNC in destructive mechanical stress through its elastic properties [67,68] has been suggested in aortic tissue [69].

The most well-documented role of TNC may be in the regulation of immune/inflammatory reactions in various pathologies, including cancer stroma [14,15]. In the inflammatory phase after MI, cardiomyocyte necrosis results in the release of the molecular signals, damage-associated molecular patterns (DAMPs) [70,71,72], which trigger inflammation-driven fibrotic responses [73,74]. TNC functions as a DAMP and is one of the ligands for TLR4 [72,75,76]. TNC induces the synthesis of proinflammatory cytokines through

TLR4 [55,57,75,76,77,78,79] and up-regulates the expression of MMPs in various types of cells, including macrophages [77,80].

Monocytes/macrophages are the main players in post-infarct inflammation [81]. A heterogeneous population of macrophages have been shown to infiltrate infarcted lesions [82,83,84,85,86], and are broadly classified as M1 or M2 macrophages [87]. M1 macrophages are associated with pro-inflammatory responses and are referred to as “classically activated macrophages”, while M2 macrophages, or “alternatively activated” macrophages, exhibit an anti-inflammatory and pro-regenerative phenotype [88]. On days 1–3 post-MI, M1-like macrophages primarily infiltrate the injured myocardium, drive inflammation, and remove damaged tissue. On approximately days 5–7 post-MI, infiltrating macrophages predominantly exhibit the M2-like phenotype [57,89]. A well-controlled switch from M1 to M2 is necessary for the appropriate resolution of inflammation and the facilitation of tissue healing [90].

**Figure 1:**
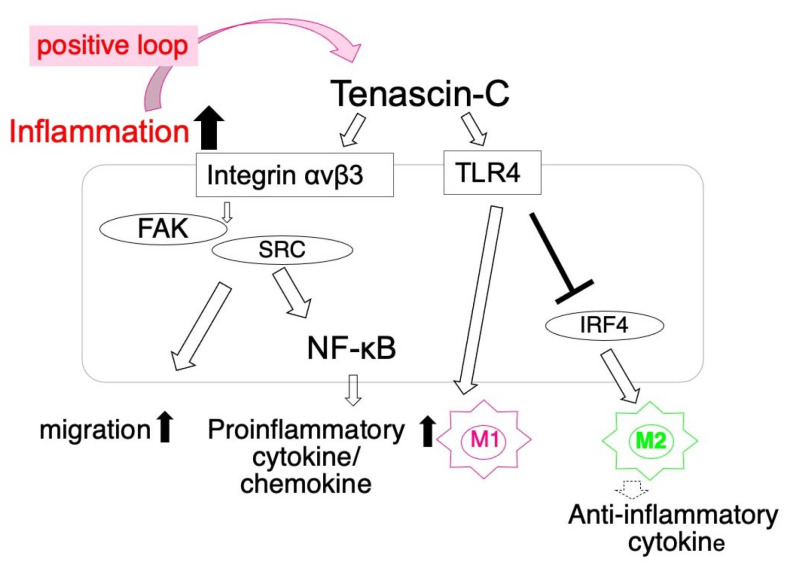
Schematic illustration showing a hypothetical mechanism by which tenascin-C enhances proinflammatory responses of macrophages.

TNC enhances the pro-inflammatory phenotype of macrophages. Its deletion inhibited the expansion of M1, but enhanced that of M2 in a MI mouse model. In vitro, TNC/TLR4 enhanced the M1 macrophage polarization of bone marrow-derived macrophages, but inhibited the up-regulation of the M2 macrophage marker by suppressing interferon regulatory factor 4 (IRF4) [57]. TNC also accelerated the migration and up-regulated the synthesis of proinflammatory cytokines/chemokines via integrin αvβ3 of peritoneal macrophages [56]. Furthermore, proinflammatory cytokines up-regulated the expression of TNC, which may amplify inflammatory responses by creating a positive feedback loop [13,91] (Figure 1). TNC also induced the activation of inflammasomes through TRL4 in epicardial cells [92]. The overexpression of TNC in the mouse heart was shown to up-regulate the expression of proinflammatory cytokines/chemokines and increase mortality rates during the acute stage after MI [19]. Collectively, these findings indicate that TNC enhances proinflammatory responses during the acute stage after infarction.

Although the TNC/TRL4 axis drives inflammation, it also plays an important role in tissue repair [93]. Myocardial repair is primarily dependent on the recruitment and activation of fibroblasts/myofibroblasts because of the limited regenerative ability of cardiomyocytes. Myofibroblasts are specialized fibroblasts that express smooth muscle cell-specific proteins, such as α-smooth muscle actin (SMA), generate contract forces to minimize injured sites, and synthesize collagen to replace the tissue defect [94].

TNC may facilitate the recruitment of myofibroblasts in various tissues [95]. In a MI model of TNC knockout mice, the appearance of myofibroblasts in the injured heart was later than in wild-type mice [61]. In vitro, TNC accelerated the migration of fibroblasts, their conversion to myofibroblasts, as well as contractile force generation and collagen synthesis by fibroblasts [39,61,92,93], at least partly via the integrin αvβ1/TGF-β/smad2/3 cascade [96]. In terms of fibroblasts/myofibroblasts, TNC promoted myocardial repair by facilitating healing activities.

### 4.2. Post-Infarct Ventricular Remodeling and TNC

The most important clinical issue in patients with MI is post-infarct ventricular remodeling, which refers to the progressive dilatation of ventricles associated with systolic dysfunction weeks or months after infarction.

Previous clinical studies suggested that TNC contributes to the progression of adverse ventricular remodeling. MI patients with high serum levels of TNC in the acute stage were found to be at an increased risk of ventricular dilatation in the chronic stage and had a poor long-term prognosis [59,97]. Mouse model experiments revealed that post-infarct remodeling was attenuated in TNC knockout mice [51,54,57], suggesting that TNC is a harmful molecule that has a negative impact on ventricular remodeling. Dilatation of the ventricular chamber in the acute stage after infarction is explained by the rearrangement (slippage) of cardiomyocytes and degradation of the interstitial matrix by MMPs (reviewed in [98,99]). As a de-adhesion molecule, TNC may augment slippage [25,30]. Furthermore, the over-activation and inappropriate resolution of inflammation by the proinflammatory functions of TNC may also augment tissue destruction. However, difficulties are associated with elucidating the mechanisms by which TNC exacerbates the progression of post-infarct ventricular remodeling in the chronic stage because its expression is limited in the acute stage. A mouse model of post-infarct ventricular remodeling demonstrated that wild-type mice showed significantly poorer function and greater dilatation of the ventricles 3 months after MI than TNC-KO mice; however, the expression of TNC in the wild type had disappeared by this time point. Furthermore, no significant differences were observed in IL-6, TNF-α, or CCL2 levels between wild-type and TNC-KO mice at this time point [69]. Ventricular remodeling is a complex mechanism. The dysregulated process from inflammation to tissue repair during the acute stage may result in physically weak scar tissue at the healed stage, and TNC may at least partly, but significantly, contribute to adverse post-infarct ventricular remodeling.

## 5. Hypertensive Cardiac Fibrosis and TNC

Cardiac fibrosis is defined as the excessive deposition of collagen fibers in the myocardium and is observed in virtually all pathological hearts [86,100], such as MI, pressure overload [101], diabetes mellitus [102], hypertrophic cardiomyopathy [103], and DCM [104,105].

It is generally classified into 2 types: replacement/reparative and reactive fibrosis [106]. Scar formation after MI, discussed in the previous section, is a typical example of replacement/reparative fibrosis. In contrast, reactive fibrosis describes an increase in perivascular and interstitial collagen fibers in the absence of the significant loss of cardiomyocytes. This type of fibrosis is often observed in pressure or volume overload, aging, and diabetes mellitus. However, it is practically difficult to distinguish between reactive interstitial reactive fibrosis and replacement fibrosis caused by small infarct lesion low-flow ischemia in the advanced stages of myocardial diseases. In any case, similar molecular mechanisms may mediate fibrosis.

Previous findings suggested that ‘low-grade (smoldering) chronic inflammation’ is involved in the progression of reactive fibrosis in the pressure-overloaded hypertrophic heart [107,108,109]. This type of inflammation is characterized by the recruitment of immune cells, particularly macrophages, at the perivascular area. One of the well-studied neurohumoral cascades that link mechanical stress and inflammation/fibrosis is the renin/aldosterone/angiotensin system (RAAS). An angiotensin II (ANG II) or aldosterone infusion model as well as a transverse aortic constriction (TAC) model are often used to investigate the cellular and molecular mechanisms underlying reactive fibrosis.

In the ANG II-induced hypertensive hearts of mice, collagen fibers at the perivascular area increase, extend between individual myocytes, and are associated with macrophage infiltration. Interstitial cells synthesize TNC at perivascular lesions, which is then deposited at the vicinity of macrophages [28,56]. TNC stimulates macrophages in a paracrine manner via the integrin αVβ3/FAK/Src/Nuclear Factor-κB axis to facilitate the migration and expression of proinflammatory cytokines, including IL-6. IL-6 is also known as a pro-fibrotic cytokine that stimulates fibroblasts to synthesize collagen, leading to fibrosis [56]. Proinflammatory cytokines released from macrophages up-regulate the synthesis of TNC by cardiac fibroblasts in a paracrine manner, such that TNC may facilitate fibrosis by promoting inflammation with a positive feedback loop (Figure 1). Furthermore, TNC may directly stimulate fibroblasts via integrin αVβ1/TGFβ/smad2/3 [96] and PDGF-A-B/PDGF-receptor β [28] to augment reactive fibrosis.

In support of this schema of inflammation/fibrosis, a previous study using BALB/c mice with the knockout of TNC showed that ANG-II induced inflammation and fibrosis were less severe than in wild-type mice [56]. The genetic background is important in immune responses. BALB/c mice favor Th2 responses, whereas C57BL/6 mice show a preference for Th1 responses [110]. This difference may influence inflammatory/fibrotic responses in disease models and regulatory mechanisms. However, similar findings were observed in the TAC model using C57BL/6 mice with the knockout of TNC, suggesting proinflammatory and profibrotic roles for TNC [23,52,111]. In contrast, fibrosis was exacerbated by enhanced inflammation in the TAC model using C57BL/6 mice with the knockout of TNC [53].

This may be explained by the context dependency of TNC; it sometimes exhibits opposing functions depending on the experimental design, which contributes to the difficulties associated with clarifying the functions of this molecule.

## 6. Myocarditis

### 6.1. Acute Myocarditis and TNC

Myocarditis is an inflammatory condition in the myocardium that is histopathologically defined by inflammatory cell infiltration adjacent to cardiomyocytes and is classified as follows based on the predominant type of infiltrating cell: lymphocytic, eosinophilic, giant cell, or granulomatous [112].Lymphocytic myocarditis is the most common type and is characterized by the predominant infiltration of CD4- and CD8-positive T lymphocytes associated with CD68-positive macrophages [113]. However, the cell profile of infiltrates may vary according to the stage of disease [114].

Myocarditis may be caused by various infectious agents, exposure to drug treatments, and immune disorders [115]. Immune checkpoint inhibitor-related myocarditis has recently been attracting increasing attention in onco-cardiology [116,117].

Viral infection is a major cause of acute lymphocytic myocarditis in the developed world [118]. Cardiotropic viruses, such as coxsackievirus B3 (CVB3) and adenoviruses, have been implicated as a trigger of myocarditis. However, vasculotropic viruses, such as parvovirus B19, and lymphotropic viruses, including human herpesvirus 6, in patients with myocarditis are now being more frequently reported [115,119]. Severe acute respiratory syndrome coronavirus 2 (SARS-CoV-2) may cause cardiomyocyte injury via immune responses or macro/microvascular thrombi rather than by direct virus-mediated cytotoxicity [115,119]; however, the classic type of acute lymphocytic myocarditis is rare [120,121].

Mouse models of CVB3 infection have been used to investigate the pathogenesis of viral myocarditis [122,123].Within 3–4 days of inoculation, CVB3 enters cardiomyocytes by binding to the coxsackievirus-adenovirus receptor and directly causes cytotoxicity.

Cell injury activates innate immunity via pattern recognition receptors, such as TLRs, and various proinflammatory cytokines are released. Antigen-specific responses in adaptive immune cells, mostly T cells, then eliminate the virus and infected cells for up to 14 days (reviewed in [124]). Furthermore, as a potential consequence of expanding self-antigen exposure from virus-infected cardiomyocytes or epitope cross-reactivity, CVB3 infection may increase the number of autoreactive CD4+ T cells for multiple antigens, which contributes to the development of myocarditis [125,126].

The myosin heavy chain α isoform (MyHC-α) is a major cardiac autoantigen. MyHC-α-reactive T-cell numbers were previously reported to be markedly increased in myocarditis patients [127]. MyHC-α immunization with immune adjuvants or an injection of MyHC-α-loaded dendritic cells (DCs) induced autoimmune myocarditis in mice [128,129]. Therefore, virus-triggered immune reactions may be the principle cause of cardiomyocyte injury rather than direct virus-mediated cell injury [115]; however, the pathophysiology of myocarditis may vary depending on the causative virus.

**Figure 2:**
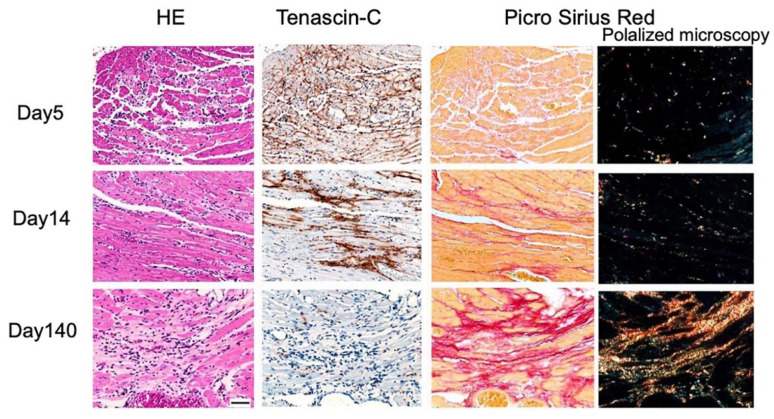
Sequential changes in the expression of TNC in a mouse myocarditis model of coxsackievirus B3 infection. The expression of TNC is observed in the early stage, on day 5 of inoculation. The expression of TNC persists during active inflammation and disappears in the healed phase when mature collagen fibers are formed. Bar, 50 µm.

In a mouse CVB3 myocarditis model, the expression of TNC was detected in the early stage after the inoculation of the virus (Figure 2).

Recent studies suggested the involvement of TNC in the pathophysiological mechanism of viral infection. Mills et al. showed that a viral infection may directly induce the expression of TNC [130]. A synthetic viral mimic of human rhinoviruses induced TNC and TN-C-rich small extracellular vesicles, such as exosomes released from bronchial epithelial cells, via a stimulation with TLR3.

Viral infection-induced TNC may be involved in defense mechanisms against viral infection. TNC in breast milk binds to the HIV-1 envelope via fibrinogen-like globe (fbg) and fibronectin-type III (fn) domains, which neutralize HIV [131,132,133]. In contrast, recent findings indicated that TNC was delivered to the cells of distant organs in COVID-19 patients through plasma exosomes and may trigger pro-inflammatory cytokine signaling as a contributory factor to the cytokine storm [134]. Furthermore, TNC may modify the disease state of viral infection by interacting with other pathogens. For example, TNC binds to Streptococcus pyogenes (group A Streptococcus; GAS) and may increase GAS colonization, leading to GAS-influenza-A superinfection [135].

In mouse CVB3 myocarditis, the expression of TNC persisted during active inflammation after the elimination of the virus from the tissue [136] and disappeared in the healed phase (Figure 2), which was similar to that observed in an experimental mouse autoimmune myocarditis model [26]. The up-regulated expression of TNC at focal active sites of inflammation has been reported in endomyocardial biopsy specimens from human myocarditis patients [27], including immune checkpoint inhibitor-induced myocarditis [137]. Cross bone marrow transplantation experiments in mice revealed that the major source of TNC was residential fibroblasts in the heart and not bone marrow-derived inflammatory cells [13,111].

Locally synthesized TNC plays an important role in regulating immune/inflammatory responses in myocarditis. TNC may trigger inflammation [75] as a DAMP molecule and amplify the inflammatory responses of macrophages by creating a positive feedback loop [13,91], as discussed in an earlier section. Furthermore, TNC activates DCs to generate pathogenic autoreactive T cells and forms an important link between innate and acquired immunity [55,138]. DCs stimulated by TNC have been shown to produce various proinflammatory cytokines, including IL-6, which, in turn, induce naïve CD4+ T cells to differentiate into Th17 cells. Th17 cells are closely associated with autoimmunity and play a major role in myocarditis [139,140].

TNC also exerts diverse effects on lymphocytes (reviewed in [141]). It has been shown to support lymphocyte rolling [142], lymphoid progenitor cell homing, and peripheral T-cell migration [143,144], and also promotes T-cell activation and polarization [75,145,146,147,148], but inhibits the proliferation of CD4+ T cells [141].

The deletion of TNC may attenuate the severity of myocarditis by reducing Th17 cell infiltration in the mouse heart [55], suggesting that TNC adversely affects the status of myocarditis; however, it may also have an immunosuppressive function depending on both the inflammatory context and splicoform expressed [15,149].

### 6.2. Chronic Myocarditis, Inflammatory DCM(Idcm), Heart Failure and TNC

Acute inflammation is a protective response to infection and is typically self-limiting. However, the failure to clear viruses from the heart or dysregulation of the immune system may result in chronic myocarditis. Although the mechanisms underlying the transition from acute to chronic inflammation currently remain unclear, TNC may play a role in the persistence of inflammation [75]. The expression of TNC has been detected in a mouse model of chronic myocarditis induced by recombinant Bacille Calmette-Guérin (BCG) expressing a cardiac autoantigen [150] as well as in endomyocardial biopsy specimens from patients with chronic myocarditis [151]. Prolonged inflammation in chronic myocarditis induces progressive tissue destruction, eventually resulting in the clinical phenotypes of DCM with severe heart failure. DCM is a clinically defined heterogeneous group of myocardial diseases with left ventricular dilatation and contractile dysfunction [152]. DCM patients often have genetic mutations [153], although some cases may be attributed to chronic myocarditis [113]. Chronic myocarditis needs to be distinguished from other types of DCM because immunosuppressive agents may control disease progression in chronic myocarditis and eliminate the need for heart transplantation, whereas gene mutations cannot be treated by immunosuppression.

In addition to recognizing the importance of inflammation in the progression of heart failure, the term ‘iDCM’ is often used for a subgroup of DCM associated with inflammation. ‘Inflammation’ is detected in endomyocardial biopsy samples from iDCM patients, who have a poorer prognosis than DCM patients without inflammation [154,155]. However, it is important to note that iDCM and chronic myocarditis may not be synonymous. In several cases of DCM, gene mutations in the compositional elements of cardiomyocytes may contribute to vulnerability to stress in the failing heart, resulting in cell injury or death, which triggers innate immune responses. Although inflammation is an essential response during tissue repair, inflammatory mediators released during reactive inflammation in DCM hearts may further aggravate heart failure, resulting in a vicious cycle of inflammation [113]. Therefore, iDCM may be part of this pathological condition and the type of inflammation may differ from that of narrowly defined myocarditis predominantly mediated by acquired immunity, as discussed above. TNC was previously shown to be expressed in iDCM hearts and was associated with worse LV remodeling and long-term outcomes in DCM [156]. Since TNC amplifies inflammatory responses by creating several positive feedback loops in various pathophysiologies, it may enhance the vicious cycle in iDCM.

## 7. Clinical Application of TNC

TNC has potential as a diagnostic marker and target for the molecular imaging of inflammation based on its specific expression, which may facilitate the differentiation of myocarditis from DCM.

Immunostaining for TNC is useful for a precise diagnosis of active inflammation in biopsy samples of the myocardium from myocarditis and DCM patients [27,156,157]. A combination with molecular imaging for TNC may reduce the sampling errors of endomyocardial biopsy by clarifying the target site for biopsy in the heart. To date, several TNC-specific monoclonal antibodies or antibody fragments (scFv) have been developed [158,159] and are undergoing evaluations for inflammation imaging in the heart [160,161,162,163]. Furthermore, serum/plasma TNC may be a practical diagnostic biomarker. Elevated levels of TNC in the blood may reflect the up-regulated expression of TNC in the inflammatory lesions of DCM and predict worse left ventricular remodeling and long-term outcomes [156,164,165,166].

Similarly, TNC is applicable as a biomarker to detect acute and chronic rejection after human cardiac transplantation in association with inflammation [167].

A recent study reported that serum TNC may predict an increased risk of cardiovascular disease in patients with type 2 diabetes associated with chronic low-grade inflammation [168]. It is also important to note that TNC may also be useful for evaluating the disease condition of heart failure with a preserved ejection fraction (HFpEF) [169]. HFpEF is a leading cause of morbidity and mortality throughout the industrialized world. Although HFpEF is a heterogeneous group of heart diseases, obesity and type 2 diabetes mellitus are comorbidities in most patients. A recent study proposed that HFpEF is a multisystem disorder involving up-regulated immune and inflammatory signaling [170].

Serum TNC may be useful for the management of patients with arrythmia because fibrosis is considered to play a central role in stabilizing the re-entrant drivers that maintain arrhythmia [171]. TNC levels were found to be higher in patients with malignant arrythmia than in those with benign arrythmia [172]. In patients with atrial fibrillation, TNC levels in the left and right atria correlated with the severity of atrial dilatation [173].

An increasing number of studies have examined the clinical utility of serum TNC levels in patients with various heart diseases (reviewed in [149,174,175]), such as non-compaction/hypertrabeculation [176], hypertrophic cardiomyopathy [177], heart failure with ischemic heart disease, Kawasaki disease [178], rheumatic heart disease, and congenital heart disease in pediatric patients [179,180].

Many studies demonstrated that high serum TNC levels predicted adverse ventricular remodeling and increased the risk of death and major adverse cardiovascular events, suggesting that TNC is not only a marker for tissue damage and inflammation, it is also a ‘remodeling’ marker. In contrast, decreased TNC levels may be useful for evaluating reverse remodeling [181]. A recent study demonstrated that decreased TNC levels reflect the effectiveness of intravenous immunoglobulin therapy for Kawasaki disease and are useful as an indicator for assessing whether inflammation has decreased [182].

Moreover, the combination of serum TNC levels and plasma BNP levels is a stronger predictor than either biomarker alone in AMI and DCM [97,165]. This may be explained as follows: while BNP is secreted from stressed cardiomyocytes, TNC is released by activated interstitial fibroblasts. Interstitial fibroblasts may play a major role in the reconstruction of the injured heart because the regenerative ability of cardiomyocytes is very limited. Therefore, a combination of the two biomarkers may contribute to a more precise assessment of the whole heart by evaluating cardiomyocyte stress and the interstitial repair reaction.

Other matricellular proteins, such as galectin-3 [183], periostin [184], and osteopontin [185,186] have also been proposed as cardiovascular biomarkers. Galectin-3 is recommended by the 2017 Guidelines of the American Heart Association for the risk stratification and prognostic evaluation of patients with heart failure [187]. These matricellular proteins are up-regulated by tissue injury and inflammation and show a similar expression pattern to that of TNC. They sometimes localize with TNC and may interact. Periostin [188] and galectin-3 [189] directly bind to TNC, appear to regulate the expression levels of each other [33], and play a role in the regulation of inflammation and fibrosis. Furthermore, osteopontin and TNC have been shown to share some receptors and sometimes exert opposite effects [190]. They may regulate tissue inflammatory fibrotic responses by cooperating or counterbalancing each other. Therefore, each biomarker may reflect different cellular activities during myocardial tissue remodeling. The combination of these matricellular biomarkers may be useful for the more precise stratification of myocardial disease conditions and selection of appropriate therapies.

## 8. Conclusions

TNC may be a feasible diagnostic biomarker and target for the molecular imaging of inflammation because of its specific expression associated with inflammation, which may contribute to the stratification of patients with heart diseases and the selection of appropriate therapy.

Experimental studies using TNC-knockout and -overexpressing mice suggest that TNC is a noxious molecule in the heart because it aggravates inflammation/fibrosis in most cases. However, TNC has diverse functions and, thus, may exert both harmful and protective effects in the heart. The inhibition of the proinflammatory functions of TNC with appropriate timing may provide novel strategies that prevent the progression of heart failure.

## Data Availability

Not applicable.
